# A special issue on “New technologies in parasitology”

**DOI:** 10.1007/s00436-022-07486-8

**Published:** 2022-03-18

**Authors:** Klaus Brehm, Anja Taubert, Christoph G. Grevelding

**Affiliations:** 1 University of Würzburg, Würzburg, Germany; 2 Justus Liebig University Giessen, Giessen, Germany

## Preface

Recent years have documented a revolutionary change in research on parasitic organisms. The development of pioneering technologies has allowed answering fundamental questions of parasite biology, from the perspective of basic research but also with respect to applied aspects. Comprehensive molecular, cellular, and biochemical information is available today, which includes a wealth of information on genomes, transcriptomes, proteomes, and glycomes. This has not only revolutionized parasitology research but significantly advanced parasitological diagnostics. Flanked by improvements in parasite ex vivo culturing and maintenance, and promoted by groundbreaking methods for imaging and the functional analyses of genes of interest within their genomic settings, we have gained new and spectacular insights into parasite biology and host-parasite interactions. This unprecedented upswing has lifted parasitology to a new level, and it facilitates better understanding of parasites and the principles of parasitism. Thus, new strategies in fighting parasites may be achieved in the medium term. The latter is of utmost importance considering the lack of anti-parasitic vaccines, the alarmingly limited set of effective drugs available for humans and animals, and increasing reports on drug resistance.

